# Geographical inequalities in lung cancer management and survival in South East England: evidence of variation in access to oncology services?

**DOI:** 10.1038/sj.bjc.6600831

**Published:** 2003-04-01

**Authors:** R H Jack, M C Gulliford, J Ferguson, H Møller

**Affiliations:** 1 Department of Public Health Sciences, King's College London, UK; 2 Lambeth Southwark and Lewisham Health Authority, 1 Lower Marsh, London SE1 7NT, UK; 3 Thames Cancer Registry, King's College London, UK

**Keywords:** lung cancer, radiotherapy, chemotherapy, surgery, survival, health services

## Abstract

This study aimed to determine whether the management and survival of patients with lung cancer varied among 26 health authorities in South East England. The Thames Cancer Registry identified patients diagnosed with lung cancer (ICD-10 codes C33–C34) between 1995 and 1999. After excluding death certificate only patients, 32 818 (81%) patients were analysed. The proportions of patients receiving active treatment varied among health authorities between 5 and 17% for non-investigative surgery, 4 and 17% for any chemotherapy, 8 and 30% for any radiotherapy and 15 and 42% for any active treatment. One-year patient survival ranged from 11 to 34%. There was evidence of health authority level variation even after adjusting for case mix. Patients whose first hospital attendance was at a radiotherapy centre were more likely to receive active treatment (OR 1.72, 95% CI 1.21–2.46), chemotherapy (1.38, 1.06–1.79) or radiotherapy (1.86, 1.28–2.71). There was some evidence that patients whose first hospital attendance was at a radiotherapy centre survived longer. This study shows there is geographical inequality in the treatment given to lung cancer patients and patient survival in South East England. There was some evidence to suggest that these inequalities might be explained by variations in access to oncology services. Future studies should investigate the pathways and barriers to specialist care in this condition.

Cancer is one of the main causes of death in Britain, and reducing cancer mortality is a recognised national priority (Department of Health, 1998a). There is great concern that survival rates with cancer are relatively low in Britain when compared with Europe (Janssen-Heijnen *et al*, 1998), and survival rates in Europe are lower than in North America (Gatta *et al*, 2000). The management of lung cancer is of particular concern because it is the most common cancer, it is associated with older age and social deprivation, survival rates are very low and treatment may often be suboptimal (Department of Health, 1998b). However, there -is now good evidence that active treatment, either with surgery or radiotherapy for non-small cell lung cancer, or with radiotherapy or chemotherapy for small cell lung cancer, may prolong survival and should not be denied to patients who might benefit (Brown *et al*, 1996; Fergusson *et al*, 1996; Muers and Haward, 1996).

A number of reports have identified problems with the care of patients with lung cancer. Patients presenting with symptoms suggestive of lung cancer often experience delays at every stage of the referral process. Billing and Wells (1996) found that the median delay experienced by patients referred for surgery was 109 days, including 1 month before initial referral to a specialist, and 2 months before subsequent referral to a surgeon. Melling *et al* (2002) found variations in the investigative procedures and surgery received by patients with different modes of presentation to hospitals (whether they were referred by their GP with or without a chest X-ray, or presented themselves to the Accident and Emergency Department). Recommended waiting times have been shown to be achievable for most patients using a multidisciplinary team (Deegan *et al*, 1998). Studies show around 70% of patients receive a histological diagnosis, although this figure decreases with age to 55% of patients aged 75–84 years (Kesson *et al*, 1998) and is only 48% in patients not reviewed by a respiratory specialist (Brown *et al*, 1996). A failure to evaluate the stage of the disease accurately may have adverse effects. Patients may be denied effective treatment if it is assumed that disease is widespread at presentation, but thoracotomy should be avoided in patients with unresectable disease. Some evidence suggests that the volume of cases treated may be associated with the outcome (Selby *et al*, 1996; Hillner *et al*, 2000); some studies have suggested benefits from nurse-run clinics (Department of Health, 1998c); other studies suggest that more appropriate care may be provided by specialist respiratory physicians (Brown *et al*, 1996). Kesson *et al* (1998) found that patients who were originally referred to non-respiratory physicians had a significantly longer delay before surgery.

In a study of patients living in Scotland and diagnosed with lung cancer in 1995 deprivation did not affect chances of receiving potentially curative treatment, but the most deprived quintile of residents were significantly less likely to survive 3 years compared to the least deprived quintile (Gregor *et al*, 2001). The health board of residence was found to be associated with receiving thoracic radiotherapy for those with non-small cell or unknown type of lung cancer (Erridge *et al*, 2002).

Against this background, the English Department of Health has developed a strategy to improve cancer services in England (Department of Health, 1995). The strategy is establishing subregional cancer networks whose aim is ‘to provide the means by which healthcare professionals, treating cancer across an area can jointly agree, implement and monitor the most appropriate patterns of care for their patients’ (Northern and Yorkshire Cancer Information Service, 2000). In June 1998, the Department of Health published guidance on commissioning services for lung cancer under the title *‘Improving Outcomes in Lung Cancer’* (Department of Health, 1998b,1998c). The NHS Cancer Plan was launched in September 2000 and aimed to tackle inequalities in quality of care and treatment (Department of Health, 2000).

The present population-based study aimed to determine whether there were variations in case mix, and inequalities in case management, for lung cancer among 26 health authorities in South East England from 1995 to 1999. We also wanted to find out whether, after adjusting for case mix, there were area or health service characteristics that were associated with more, or less, favourable treatment patterns and with patient survival.

## MATERIALS AND METHODS

### Data

The Thames Cancer Registry supplied information on lung cancer patients (ICD-10 codes C33–C34) resident in 26 health authorities in South East England (Essex, Hertfordshire, London, Kent, Surrey and Sussex), diagnosed between 1995 and 1999. Data on sex, age at diagnosis, date of diagnosis, date of death (if applicable), basis of diagnosis, histological type, stage of tumour, treatment and health authority were also available. The basis of diagnosis was either ‘clinical’ or ‘histological’ and the histological category was further divided into ‘small cell’, ‘non-small cell’ and ‘other and unspecified’ types. The non-small cell category was made up of squamous cell (55%), adenocarcinoma (28%), large cell (7%) and other non-small cell carcinomas (10%). Standard TNM staging was available for very few patients, so stage was classified as ‘localised’, ‘direct extension’, ‘local lymph nodes’, ‘metastases’ or ‘not known’. These categories have been shown to be predictive of survival for lung cancer patients (Thames Cancer Registry, 2000) and have been used in previous studies (Gulliford *et al*, 1993a). Indicator variables were specified to identify those subjects who received any radiotherapy, any chemotherapy, any non-investigative surgery (defined as either pneumonectomy or lobectomy) or any active treatment (surgery, radiotherapy or chemotherapy).

From the dates of diagnosis and death, variables were created indicating whether the patient had survived 1 or 3 years after diagnosis. Survival times were censored on 31 December 1999. This was the last year for which complete data were available. The age standardised lung cancer incidence rate was calculated for each district with reference to the European standard population. The Townsend deprivation score was obtained from the Public Health Common Data Set (Department of Health, 2001). We also determined whether the first hospital trust visited as an inpatient or outpatient was a radiotherapy centre.

### Analysis

We initially tabulated case mix and treatment variables by year of study. For each variable, we estimated the percentage of patients in each category by health authority. Data are presented as the median and range in order to document variation between health authorities.

The data had a hierarchical structure with individual patients at level one, and health authorities (or hospitals) at level two. Level one units (patients) were nested within level two units (hospitals or health authorities). Random effects (multilevel) logistic regression models were fitted with health authority or hospital as a random effect. This allowed for correlation in patient outcomes within health authorities or hospitals. Analyses were performed using the multilevel modelling package MLwiN (Rasbash *et al*, 2000). Dependent variables included whether the patient received any non-investigative surgery, any radiotherapy, any chemotherapy or any active treatment. As explanatory variables the patient's gender, age at diagnosis (by 10 year group), basis of diagnosis, histological type and tumour stage were fitted as fixed effects. Area and hospital characteristics were also fitted simultaneously as fixed effects. Similar models were used to investigate variation in 1- and 3-year survival between health authorities after adjusting for treatment as well as case mix. When area and hospital characteristics were included in the analysis, hospital first attended was fitted as the level two variable instead of the health authority. To assess whether completeness of information was influencing the results the proportion of cases that were registered only on the basis of a death certificate (DCO cases) in each health authority was also fitted as a fixed effect.

## RESULTS

There were 40 540 subjects with lung cancer registered in the 26 health authorities between 1995 and 1999. After excluding 13 cases because of inconsistencies in the data and 7 709 ‘death certificate only’ (DCO) registrations, 32 818 (81%) cases were analysed. The percentage of DCO registrations decreased during the time of the study from 25% in 1995 to 12% in 1999. The proportion of DCO registrations varied from 10 to 28% in different health authorities.

Table 1

**Table 1:** Frequencies (column %) of characteristics of non-DCO lung cancer patients diagnosed in South East England 1995–1999

	**Year of diagnosis**											
	**1995**		**1996**		**1997**		**1998**		**1999**		**All years**	
	***n***	**%**	***n***	**%**	***n***	**%**	***n***	**%**	***n***	**%**	***n***	**%**
*Sex*												
Male	4007	64	3955	63	4122	63	4336	62	4165	61	20 585	63
Female	2298	36	2304	37	2380	37	2615	38	2636	39	12 233	37
												
*Age at diagnosis groups (years)*												
<35	12	0	18	0	9	0	17	0	15	0	71	0
35–44	96	2	78	1	73	1	87	1	82	1	416	1
45–54	397	6	425	7	448	7	420	6	440	6	2130	6
55–64	1047	17	1057	17	1101	17	1154	17	1152	17	5511	17
65–74	2275	36	2327	37	2380	37	2412	35	2278	33	11 672	36
75–84	1989	32	1855	30	2016	31	2239	32	2183	32	10 282	31
85+	489	8	499	8	475	7	622	9	651	10	2736	8
												
*Basis of diagnosis*												
Clinical	1308	21	1189	19	1317	20	1360	20	1335	20	6509	20
Histological	4684	74	4816	77	4895	75	5277	76	5104	75	24 776	75
Other/not known	313	5	254	4	290	4	314	5	362	5	1533	5
												
*Histological type*												
Small cell	863	14	879	14	837	13	899	13	865	13	4343	13
Non-small cell	3306	52	3294	53	3391	52	3783	54	3851	57	17 625	54
Other/not known	2136	34	2086	33	2274	35	2269	33	2085	31	10 850	33
												
*Stage*												
Localised	1706	27	2078	33	2412	37	2608	38	2637	39	11 441	35
Direct extension	254	4	299	5	308	5	345	5	278	4	1484	5
Local lymph nodes	205	3	204	3	216	3	212	3	184	3	1021	3
Metastases	1776	28	1887	30	1830	28	2210	32	2240	33	9943	30
Not known	2364	37	1791	29	1736	27	1576	23	1462	21	8929	27
												
*Deprivation quartile*												
1 (least deprived)	1715	27	1769	28	1808	28	1999	29	1911	28	9202	28
2	1739	28	1689	27	1821	28	1915	28	1910	28	9074	28
3	1292	20	1277	20	1313	20	1322	19	1329	20	6533	20
4 (most deprived)	1559	25	1524	24	1560	24	1715	25	1651	24	8009	24
												
*First hospital visited a*												
*radiotherapy centre*	1882	30	2082	33	2137	33	2236	32	2203	32	10 540	32
												
Total	6305		6259		6502		6951		6801		32 818	

shows patient characteristics by study year. The proportion of female patients significantly increased over time (test for trend *P*=0.004) from 36 to 39%. The proportion of patients aged 64 and under remained approximately constant, while the percentage aged 75 years and over increased. A histological basis of diagnosis was recorded for about 75% of patients throughout the study. The percentage of patients with non-small cell lung cancer increased over time (test for trend *P*<0.001) from 52 to 57%. More patients had their tumour staged in 1999 than previously, with most patients being classified as having either localised disease or metastases.

For the 26 health authorities, the age standardised incidence rates ranged from 37 to 67 per 100 000, and the Townsend deprivation index ranged between -5.5 (most affluent) and 12.2 (most deprived). Table 2

**Table 2:** Distribution of characteristics within health authorities. Median percentage and range of each category in health authorities, 1995–1999

	**Med %**	**Range**
Median number and range of patients	1226	621–2274
*Sex*		
Male	63	58–65
Female	37	35–42
		
*Basis*		
Clinical	20	12–30
Histological	75	67–86
Other/not known	4	1–10
		
*Histological type*		
Small cell	13	10–15
Non-small cell	53	41–62
Other/not known	35	25–45
		
*Stage*		
Localised	36	16–50
Direct extension	4	1–10
Local lymph nodes	3	1- 6
Metastases	30	24–36
Not known	25	16–54

shows the extent of variation in case mix among health authorities. The proportion with a histological diagnosis ranged from 67 to 86%. The percentage of patients with localised disease ranged from 16 to 50%, and the proportion with unclassified tumour stage ranged from 16 to 54%.

### Treatment

The extent of variation in treatment received by district of residence is shown in Table 3

**Table 3:** Median percentage and range of lung cancer patients with each treatment by health authority, 1995–1999

	**Med %**	**Range**
None registered	20	14–25
Chemotherapy only (Chem)	4	3–9
Radiotherapy only (RT)	13	6–23
Non-investigative surgery only (S)	6	4–10
Chem + RT	4	1–7
S + Chem	0	0–1
S + RT	1	0–3
S + Chem + RT	0	0–1
		
Investigative surgery	51	34–71
		
Any Chem	8	4–17
Any RT	17	8–30
Any S	7	5–12
Any treatment^a^	28	15–42
		
First hospital visited a radiotherapy centre	27	8–79

a Any treatment=any chemotherapy, radiotherapy or non-investigative surgery.

. Over all years, 15% of patients received any active treatment in one health authority compared with 42% in another. The proportion of patients receiving specific treatments also varied among health authorities, between 4 and 17% for any chemotherapy, 8 and 30% for any radiotherapy and 5 and 12% for non-investigative surgery. Non-investigative surgery was defined as pneumonectomy or lobectomy, with all other operations being classified as investigative surgery.

### Survival

Table 4

**Table 4:** Median percentage (range) of lung cancer patients surviving 1 or 3 years by health authority, including and excluding DCO patients

	**1995**		**1996**		**1997**		**1998**		**All years**	
	**Med %**	**Range**	**Med %**	**Range**	**Med %**	**Range**	**Med %**	**Range**	**Med %**	**Range**
*Excluding DCOs*										
1-year survival	28	11–33	28	14–40	28	21–32	25	20–37	28	11–34
3-year survival	11	6–14	11	6–21	—		—		11	7–17
										
*Including DCOs*										
1-year survival	19	7–27	21	9–28	22	16–27	21	17–29	22	8–24
3-year survival	7	4–11	8	4–13	—		—		8	5–12

shows the median percentages of those who survived 1 and 3 years within the health authorities, over time. Only 2 years were available for the 3-year survival and 4 years for the 1-year survival, as the data were censored on 31 December 1999. The proportion surviving 1 year ranged from 11 to 34% in different health authorities and 3-year survival ranged from 7 to 17%. When DCO patients were included in the analysis by adding a short period of survival (1 day) to all cases, these figures decreased to 8–24% for 1-year survival and 5–12% for 3-year survival.

Figure 1

**Figure 1:**
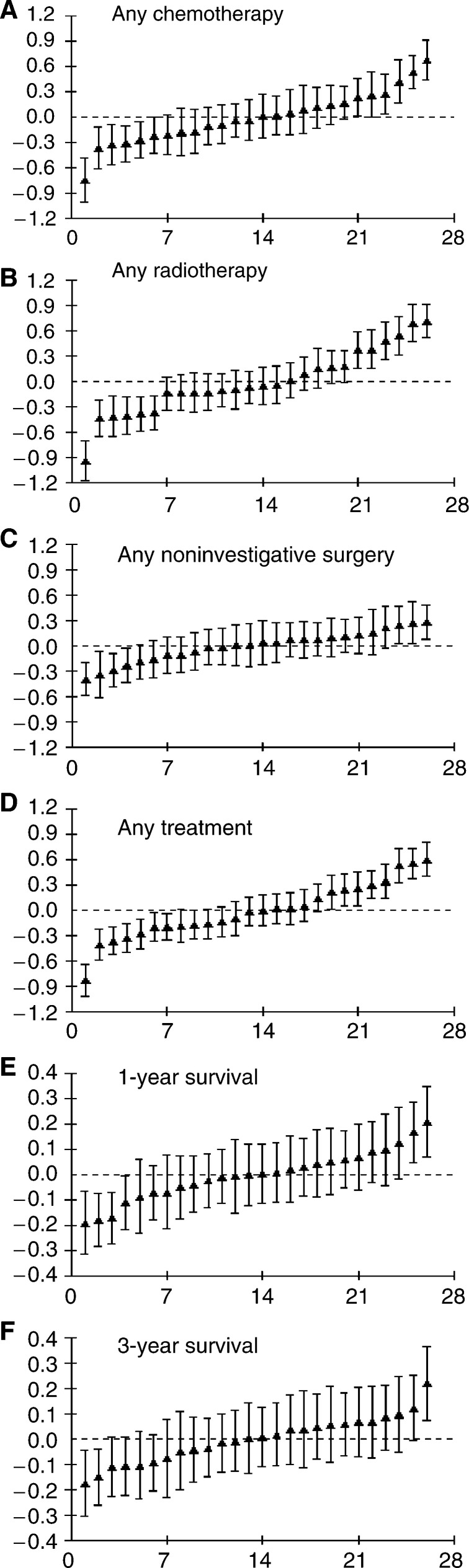
Health authority residuals and 95% confidence intervals against rank for (**A**) any chemotherapy^*^, (**B**) any radiotherapy^*^, (**C**) any non-investigative surgery^*^, (**D**) any treatment^*^ (any chemotherapy, radiotherapy or non-investigative surgery), (**E**) 1-year survival^†^, (**F**) 3-year survival^†^. ^*^Adjusted for case mix; ^†^Adjusted for case mix and treatment.

shows the standardised health authority level residuals from the multilevel models with (A) any chemotherapy, (B) any radiotherapy, (C) any non-investigative surgery, (D) any active treatment, (E) 1-year survival and (F) 3-year survival as dependent variables. The *x*-axis shows the rank of the health authorities for that outcome after adjusting for case mix (and treatment in (E) and (F)), and the *y*-axis represents the log odds of the outcome. The vertical bars show 95% confidence intervals. The figure confirms wide variation in treatment patterns among health authorities that was not explained by adjusting for case mix, and that after adjusting for case mix and treatment there was still evidence of variation in patient survival among health authorities. There was significant health authority level variation in all models, with greatest variation in the model for any radiotherapy, adjusted for case mix. As might be expected, the variation between health authorities was much smaller in the survival models than the models for treatment.

In order to evaluate whether area or hospital characteristics explained variation in treatment or survival, we included the lung cancer incidence rate, the Townsend deprivation score for the health authority and whether the first hospital trust attended was a radiotherapy centre simultaneously as additional fixed effects. Results from these analyses (Table 5

**Table 5:** Area and organisational characteristics associated with treatment and survival. Odds ratios adjusted for sex, age group, histology and stage of tumour, deprivation, lung cancer incidence, whether first hospital attended was a radiotherapy centre and hospital fitted as random effect

	**OR**	**95% CI**	***P*-value**
*Any treatment*			
First hospital visited a radiotherapy centre	1.72	1.21–2.46	0.0027
Deprivation (Townsend score)	0.99	0.97–1.00	0.1784
Age standardised incidence (per 100 000)	0.98	0.97–0.99	<0.0001
			
*Any Chemotherapy*			
First hospital visited a radiotherapy centre	1.38	1.06–1.80	0.0179
Deprivation (Townsend score)	0.96	0.94–0.98	0.0010
Age standardised incidence (per 100 000)	1.01	0.99–1.02	0.4340
			
*Any radiotherapy*			
First hospital visited a radiotherapy centre	1.86	1.28–2.71	0.0011
Deprivation (Townsend score)	1.01	0.99–1.03	0.2048
Age standardised incidence (per 100 000)	0.98	0.97–0.99	0.0034
			
*Any non-investigative surgery*			
First hospital visited a radiotherapy centre	1.02	0.63–1.64	0.9383
Deprivation (Townsend score)	0.98	0.95–1.01	0.1206
Age standardised incidence (per 100 000)	0.97	0.96–0.99	0.0005
			
*1-year survival*^a^			
First hospital visited a radiotherapy centre	1.20	0.97–1.50	0.0994
Deprivation (Townsend score)	1.01	1.00–1.03	0.1241
Age standardised incidence (per 100 000)	0.99	0.98–1.00	0.0320
			
*3-year survival*^a^			
First hospital visited a radiotherapy centre	1.18	0.97–1.43	0.0947
Deprivation (Townsend score)	1.01	0.99–1.03	0.1692
Age standardised incidence (per 100 000)	0.99	0.98–1.00	0.0446
^a^Also adjusted for active treatment.			

) showed that patients who lived in health authorities with higher lung cancer incidence rates were less likely to receive any active treatment, any radiotherapy or any non-investigative surgery. Residents of a more deprived area were less likely to receive any chemotherapy. If the first hospital visited was a radiotherapy centre, patients were more likely to receive any active treatment, any chemotherapy or any radiotherapy. There was no significant relation between survival and deprivation or whether the first hospital trust attended was a radiotherapy centre at the 5% level. However, if patients were resident in areas with high lung cancer incidence they were less likely to survive 1 and 3 years after diagnosis.

As age at diagnosis increased, patients were significantly less likely to receive any treatment (*P*=0.008), any chemotherapy (*P*<0.001) or any non-investigative surgery (*P*<0.001), or survive 1 (*P*<0.001) and 3 (*P*<0.001) years. Females were significantly more likely to receive any non-investigative surgery (*P*=0.011) and survive 1 (*P*=0.005) and 3 (*P*<0.001) years after diagnosis. Patients with metastases were significantly less likely to survive 1 (*P*<0.001) and 3 (*P*<0.001) years than those with localised disease. Treatment received was strongly linked with 1- (*P*<0.001) and 3-year (*P*<0.001) survival. All these associations were found after adjusting for area, hospital and all other case-mix variables.

Patients do not always attend a hospital in their resident health authority. Instead a cross classification occurs whereby hospitals receive patients from several health authorities, and residents of a health authority attend hospitals in various other health authorities (Goldstein, 1995; Rasbash and Browne, 2001). By fitting dummy variables MLwiN is able to model cross-classified structures and take account of clustering by both hospital and health authority. We therefore performed a sensitivity analysis by fitting multilevel models allowing for cross classification in order to evaluate whether this influenced our findings. In cross-classified models, an increase in incidence was no longer significantly associated at the 5% level with patients being less likely to receive any treatment (OR 0.98, 95% CI 0.96–1.00, *P*=0.067), any radiotherapy (OR 0.99, 95% CI 0.96–1.01, *P*=0.273), or survive 1 (OR 0.99, 95% CI 0.98–1.00, *P*=0.100) or 3 (OR 0.99, 95% CI 0.98–1.00, *P*=0.107) years. The results from cross-classified models were therefore more conservative. There was no difference in the interpretation of the radiotherapy centre models when adjusting for cross classification.

A second sensitivity analysis was performed by fitting the proportion of DCO registrations as an additional explanatory variable in the regression models. This gave small changes in odds ratios and 95% confidence intervals that did not alter the interpretation of the results. However, a unit increase in deprivation score was not significantly associated with receiving any surgery in the original model (OR 0.98, 95% CI 0.95–1.01, *P*=0.121), but after adjusting for proportion of DCO patients was significantly associated (OR 0.96, 95% CI 0.93–1.00, *P*=0025). The level of significance for the association between incidence and 3-year survival was reduced when adjusting for proportion of DCO registrations from *P*=0.045 to 0.092, although the odds ratio and 95% confidence interval remained unchanged.

## DISCUSSION

### Main findings

There is evidence of geographical inequality in the treatment given to lung cancer patients and in patient survival in South East England. These differences are not readily explained by adjusting for variations in case mix or, in the case of survival, the treatment received. Evidence of inequity is provided by the finding that residents of districts with a high incidence rate for lung cancer are less likely to receive any treatment after adjusting for deprivation, first hospital attended and case mix. A possible clue to the source of these inequalities and inequities is provided by the finding that patients who are initially seen at a radiotherapy centre are more likely to receive active treatment. However, a straightforward interpretation must be qualified for several reasons.

### Limitations of study

There were several concerns with the quality of the data. One of the main problems was the exclusion of ‘death certificate only’ patients. These had to be removed from the analysis as they included little case mix, and no treatment information. ‘Death certificate only’ cases have shorter survival times than registered patients, and therefore any survival times will be artificially high if they are excluded (Pollock and Vickers, 1994). However, cancer registry data appear to be reliable, with several studies finding high levels of agreement between registry data and patients' records (Gulliford *et al*, 1993b; Stiller, 1997). The data did not contain any information on comorbidity or performance status, which would have an impact on both treatment received and survival.

A further problem for these analyses is the likelihood that data quality and completeness would vary by health authority. There is some evidence of variation in data quality across health authorities indicated by variation in the proportion of DCO registrations (range: 10–28%) and the proportion of microscopically confirmed cases (range: 67–86%). Twelve of the health authorities had a higher than the overall 19% proportion of DCO patients, but there was no correlation between the proportion of DCO, stage not known or microscopically confirmed cases and including the DCO proportion as an additional explanatory variable had little effect on the results.

### Limitations of league tables

The limitations of performance league tables have been discussed elsewhere (Goldstein and Spiegelhalter, 1996; Adab *et al*, 2002). We have addressed these criticisms by adjusting for case mix and by fitting statistical models which allowed for the correlation of responses within areas or organisational units. We acknowledge that adjustment for case mix is necessarily incomplete and, as confounders may be misclassified, possibly biased. For these reasons, we have chosen not to identify individual health authorities in this report. The interpretation of the positive finding with respect to initial hospital of treatment is especially difficult. Patients whose condition is better, or whose disease is less severe, may be selectively referred for active treatment. This could explain why patients initially treated at a radiotherapy centre survived longer. Nevertheless, these data suggest that the best chance of obtaining active treatment is through referral to a radiotherapy centre.

### Comparison with other studies

The relation between specialisation, the volume of cases treated, and the quality and outcomes of treatment have attracted increasing attention in the study of health services. Concentrating specialist services into centres of excellence offers economies of scale, and may be associated with better case management of less common conditions which are rarely seen. However, the potential limitation of this approach is that geographical inequities in service availability will be increased, and patients who live far from specialist centres may encounter barriers to accessing services. These barriers may result either from the costs and inconvenience of travel, or from the reluctance of local professionals to make referrals to a distant centre. Campbell *et al* (2000) found increasing distance from a cancer centre was significantly associated with poorer survival for lung cancer patients in Scotland.

The empirical evidence on the potential benefits of centralisation of specialist services is contradictory. Sowden *et al* (1995) reviewed the literature relating to coronary artery surgery and found that evidence in favour of concentrating services was weak and mainly supported by small, poor–quality studies. Selby *et al* (1996) reviewed the equivalent literature for cancer care and found some evidence that patients treated at specialist centres with higher caseloads had better outcomes. Specialist hospitals had significantly better 5-year survival rates for patients aged under 75 years with breast, ovarian and rectal tumours than general hospitals in East Anglia (Stockton and Davies, 2000). If initially referred to a respiratory physician there was a significantly shorter delay before surgery in Glasgow (Kesson *et al*, 1998), and lower mortality was found when lung cancer resections were performed by specialists in South Carolina (Silvestri *et al*, 1998).

Some studies have used hospitals' patient volume as an indicator for specialisation. Bach *et al* (2001) studied lung resection procedures and found a positive association between number of procedures and patients' survival. Begg *et al* (1998) found high surgery volume was significantly linked with lower 30-day mortality for pancreatectomy, oesophagectomy, hepatic resection and pelvic exenteration operations, but not pneumonectomy. Another indication of specialisation is whether surgery takes place at a teaching hospital. This was found to be significantly associated with better 5-year survival in the USA (Bach *et al*, 2001). Teaching hospital was not represented by radiotherapy centre in this study.

### Implications of study findings

There were wide variations among districts in the use of different treatment modalities with some variation in patient survival. Studies in Yorkshire and Scotland also showed that rates of active treatment, specialist management and survival (Northern and Yorkshire Cancer Information Service, 1999) and thoracic radiotherapy (Erridge *et al*, 2002) varied by district of residence. Deprivation was related to lung cancer incidence (correlation coefficient: 0.80), and patients resident in the most deprived health authority were, on average, less likely to receive any active treatment, chemotherapy or radiotherapy. There was evidence that health authorities with higher incidence rates were less likely to provide any treatment to their residents with lung cancer.

The need to standardise cancer care and treatment has been recognised (Department of Health, 1995). The National Improving Outcomes guidance for lung cancer was published in 1998 (Department of Health, 1998b,1998c) and the NHS Cancer Plan was launched in September 2000 (Department of Health, 2000) but it is very unlikely that these initiatives will have impacted on the management of lung cancer in either 1998 or 1999. There is usually a substantial time lag from publication of guidance to its implementation and any subsequent improvement in outcomes. These analyses should therefore be repeated after a suitable interval in order to monitor changing patterns of treatment and survival.

### Conclusions

This study provides baseline data to evaluate the restructuring of cancer services in the UK. It raises several questions concerning the management of patients with lung cancer, and the organisation of services for patients with this condition. Studies are needed to find out whether current initiatives in the organisation of services for this condition are reducing inequities in access at the level of the health authority and the individual patient. Studies are also needed to determine how patients gain access to specialist services for lung cancer treatment and whether patients are referred appropriately. A better understanding of the influence of the preferences and priorities of patients and professionals in making treatment decisions is also needed.

## References

[bib1] Adab P, Rouse AM, Mohammed MA, Marshall T (2002) Performance league tables: the NHS deserves better. BMJ 324: 95–98 11786455 10.1136/bmj.324.7329.95 PMC64507

[bib2] Bach PB, Cramer LD, Schrag D, Downey RJ, Gelfand SE, Begg CB (2001) The influence of hospital volume on survival after resection for lung cancer. N Engl J Med 345: 181–188 11463014 10.1056/NEJM200107193450306

[bib3] Begg CB, Cramer LD, Hoskins WJ, Brennan MF (1998) Impact of hospital volume on operative mortality for major cancer surgery. JAMA 280: 1747–1751 9842949 10.1001/jama.280.20.1747

[bib4] Billing JS, Wells FC (1996) Delays in the diagnosis and surgical treatment of lung cancer. Thorax 51: 903–906 8984700 10.1136/thx.51.9.903 PMC472612

[bib5] Brown JS, Eraut D, Trask C, Davison AG (1996) Age and the treatment of lung cancer. Thorax 51: 564–568 8693434 10.1136/thx.51.6.564 PMC1090483

[bib6] Campbell NC, Elliott AM, Sharp L, Ritchie LD, Cassidy J, Little J (2000) Rural factors and survival from cancer: analysis of Scottish cancer registrations. Br J Cancer 82: 1863–1866 10839303 10.1054/bjoc.1999.1079 PMC2363217

[bib7] Deegan PC, Heath L, Brunskill J, Kinnear WJ, Morgan SA, Johnston ID (1998) Reducing waiting times in lung cancer. J R Coll Physicians Lond 32: 339–343 9762628 PMC9663063

[bib8] Department of Health (1995) A Policy Framework for Commissioning Cancer Services. Department of Health: London

[bib9] Department of Health (1998a) Our Healthier Nation. Department of Health: London

[bib10] Department of Health (1998b) Guidance on Commissioning Cancer Services. Improving Outcomes in Lung Cancer. The Manual. Department of Health: London

[bib11] Department of Health (1998c) Guidance on Commissioning Cancer Services. Improving Outcomes in Lung Cancer. The Evidence. Department of Health: London

[bib12] Department of Health (2000) The NHS Cancer Plan: a Plan for Investment, a Plan for Reform. Department of Health: London

[bib13] Department of Health (2001) Compendium of Clinical and Health Indicators 2000. Data Definitions and User Guide for Computer Files. September 2001 version. Department of Health and London School of Hygiene and Tropical Medicine: London

[bib14] Erridge SC, Thomson CS, Davidson J, Jones RD, Price A (2002) Factors influencing the use of thoracic radiotherapy in lung cancer – an analysis of the 1995 Scottish lung cancer audit. Clin Oncol (R Coll Radiol) 14: 219–227 12109826 10.1053/clon.2001.0046

[bib15] Fergusson RJ, Gregor A, Dodds R, Kerr G (1996) Management of lung cancer in South East Scotland. Thorax 51: 569–574 8693435 10.1136/thx.51.6.569 PMC1090484

[bib16] Gatta G, Capocaccia R, Coleman MP, Gloeckler Ries LA, Hakulinen T, Micheli A, Sant M, Verdecchia A, Berrino F (2000) Toward a comparison of survival in American and European cancer patients. Cancer 89: 893–900 10951355

[bib17] Goldstein H, Spiegelhalter DJ (1996) League tables and their limitations: statistical issues in comparisons of institutional performance. J R Statist Soc A 159: 385–443

[bib18] Goldstein H (1995) Multilevel Statistical Models. E. Arnold: London; Halsted Press: New York

[bib19] Gregor A, Thomson CS, Brewster DH, Stroner PL, Davidson J, Fergusson RJ, Milroy R (2001) Management and survival of patients with lung cancer in Scotland diagnosed in 1995: results of a national population based study. Thorax 56: 212–217 11182014 10.1136/thorax.56.3.212 PMC1758769

[bib20] Gulliford MC, Barton JR, Bourne HM (1993a) Selection for oesophagectomy and postoperative outcome in a defined population. Qual Health Care 2: 17–20 10132072 10.1136/qshc.2.1.17 PMC1055056

[bib21] Gulliford MC, Bell J, Bray F, Petruckevitch A (1993b) The reliability of Cancer Registry records. Br J Cancer 67: 819–821 8471441 10.1038/bjc.1993.149 PMC1968346

[bib22] Hillner BE, Smith TJ, Desch CE (2000) Hospital and physician volume or specialization and outcomes in cancer treatment: importance in quality of cancer care. J Clin Oncol 18: 2327–2340 10829054 10.1200/JCO.2000.18.11.2327

[bib23] Janssen-Heijnen ML, Gatta G, Forman D, Capocaccia R, Coebergh JW (1998) Variation in survival of patients with lung cancer in Europe, 1985–1989. EUROCARE Working Group. Eur J Cancer 34: 2191–2196 10070286 10.1016/s0959-8049(98)00312-8

[bib24] Kesson E, Bucknall CE, McAlpine LG, Milroy R, Hole D, Vernon DR, Macbeth F, Gillis CR (1998) Lung cancer – management and outcome in Glasgow, 1991–92. Br J Cancer 78: 1391–1395 9823985 10.1038/bjc.1998.690 PMC2063194

[bib25] Melling PP, Hatfield AC, Muers MF, Peake MD, Storer CJ, Round CE, Haward RA, Crawford SM (2002) Lung cancer referral patterns in the former Yorkshire region of the UK. Br J Cancer 86: 36–42 11857009 10.1038/sj.bjc.6600029 PMC2746535

[bib26] Muers MF, Haward RA (1996) Management of lung cancer. Thorax 51: 557–560 8693432 10.1136/thx.51.6.557 PMC1090480

[bib27] Northern and Yorkshire Cancer Information Service (1999) Cancer Treatment Policies and their Effect on Survival: Lung Cancer. NYCRIS Key Sites Study 2. NYCRIS: Leeds

[bib28] Northern and Yorkshire Cancer Information Service. http://www.yorkshire-cancer-net.org.uk Accessed: 7/12/2000

[bib29] Pollock AM, Vickers N (1994) The impact on colorectal cancer survival of cases registered by ‘death certificate only’: implications for national survival rates. Br J Cancer 70: 1229–1231 7981082 10.1038/bjc.1994.478 PMC2033695

[bib30] Rasbash J, Browne W (2001) Modelling non-hierarchical structures. In Multilevel Modelling of Health Statistics, Leyland AH, Goldstein H (eds) pp 93–105. Wiley: Chichester

[bib31] Rasbash J, Browne W, Goldstein H, Yang M, Plewis I, Healy M, Woodhouse G, Draper D, Langford I, Lewis T (2000) A User's Guide to MLwiN. Version 2.1. Institute of Education: London

[bib32] Selby P, Gillis R, Howard R (1996) Benefits from specialised cancer care. Lancet 348: 313–318 8709693 10.1016/s0140-6736(96)02482-8

[bib33] Silvestri GA, Handy J, Lackland D, Corley E, Reed CE (1998) Specialists achieve better outcomes than generalists for lung cancer surgery. Chest 114: 675–680 9743149 10.1378/chest.114.3.675

[bib34] Sowden AJ, Deeks JJ, Sheldon TA (1995) Volume and outcome in coronary artery bypass graft surgery: true association or artefact? BMJ 311: 151–155 7613425 10.1136/bmj.311.6998.151 PMC2550219

[bib35] Stiller CA (1997) Reliability of Cancer Registration Data. Eur J Cancer 33: 812–814 9291798 10.1016/s0959-8049(97)00082-8

[bib36] Stockton D, Davies T (2000) Multiple cancer site comparison of adjusted survival by hospital of treatment: an East Anglian study. Br J Cancer 82: 208–212 10638991 10.1054/bjoc.1999.0901 PMC2363169

[bib37] Thames Cancer Registry (2000) Survival analysis. In Cancer in South East England 1997: Cancer Incidence, Prevalence, Survival and Treatment for Residents of the Health Authorities in South East England, pp 75–90. Thames Cancer Registry: London

